# Epidemiological Characteristics and Genotypic Features of Rotavirus and Norovirus in Jining City, 2021–2022

**DOI:** 10.3390/v16060925

**Published:** 2024-06-07

**Authors:** Changjing Wu, Zhongyan Fu, Cuihua Xie, Jian Zhao, Feifei He, Boyan Jiao, Baihai Jiao

**Affiliations:** 1 Department of Laboratory, Jining Center for Disease Control and Prevention, Jining 272000, China; 15206766212@163.com (C.W.); xiecuihua2010@163.com (C.X.); 15668115581@163.com (J.Z.); 2 Department of Infectious Disease Control, Shandong Center for Disease Control and Prevention, Jinan 250001, China; zhongyanfu@163.com; 3 Computer Information Technology, Northern Arizona University, Flagstaff, AZ 86011, USA; fh335@nau.edu; 4 Department of Medicine, School of Medicine, University of Connecticut Health Center, Farmington, CT 06032, USA

**Keywords:** viral gastroenteritis, rotavirus, norovirus, genotypes

## Abstract

Diarrhea, often caused by viruses like rotavirus (RV) and norovirus (NV), is a global health concern. This study focuses on RV and NV in Jining City from 2021 to 2022. Between 2021 and 2022, a total of 1052 diarrhea samples were collected. Real-Time Quantitative Fluorescent Reverse Transcriptase-PCR was used to detect RV-A, NV GI, and NV GII. For RV-A-positive samples, VP7 and VP4 genes were sequenced for genotype analysis, followed by the construction of evolutionary trees. Likewise, for NV-GII-positive samples, VP1 and RdRp genes were sequenced for genotypic analysis, and evolutionary trees were subsequently constructed. Between 2021 and 2022, Jining City showed varying detection ratios: RV-A alone (excluding co-infection of RV-A and NV GII) at 7.03%, NV GI at 0.10%, NV GII alone (excluding co-infection of RV-A and NV GII) at 5.42%, and co-infection of RV-A and NV GII at 1.14%. The highest RV-A ratios were shown in children ≤1 year and 2–5 years. Jining, Jinxiang County, and Liangshan County had notably high RV-A ratios at 24.37% (excluding co-infection of RV-A and NV GII) and 18.33% (excluding co-infection of RV-A and NV GII), respectively. Jining, Qufu, and Weishan had no RV-A positives. Weishan showed the highest NV GII ratios at 35.48% (excluding co-infection of RV-A and NV GII). Genotype analysis showed that, in 2021, G9P[8] and G2P[4] were dominant at 94.44% and 5.56%, respectively. In 2022, G8P[8], G9P[8], and G1P[8] were prominent at 75.86%, 13.79%, and 10.35%, respectively. In 2021, GII.3[P12], GII.4[P16], and GII.4[P31] constituted 71.42%, 14.29%, and 14.29%, respectively. In 2022, GII.3[P12] and GII.4[P16] accounted for 55.00% and 45.00%, respectively. RV-A and NV showed varying patterns for different time frames, age groups, and regions within Jining. Genotypic shifts were also observed in prevalent RV-A and NV GII strains in Jining City from 2021 to 2022. Ongoing monitoring of RV-A and NV is recommended for effective prevention and control.

## 1. Introduction

Diarrheal disease is one of the most common illnesses in humans, affecting approximately 6 billion people globally each year and resulting in 1.3 million deaths [1,2]. It constitutes a significant global public health challenge [3,4]. The vast majority of cases of diarrhea are caused by viruses, with rotavirus (RV) and norovirus (NV) being the primary pathogens responsible for human viral gastroenteritis [5,6,7].

RV is one of the leading causative agents of diarrhea, particularly in children under the age of 5, especially in developing countries. It is a major cause of diarrhea-related deaths, accounting for approximately 450,000 deaths annually [8,9,10]. RV-A is the predominant strain of rotavirus infecting humans. Based on the VP7 and VP4 gene sequences of RV-A, it is classified into G and P genotypes. The globally prevalent genotypes include G1P[8], G2P[4], G3P[8], G4P[8], G8P[8], G9P[8], and G12P[8], among others. Different genotypes of RV-A may predominate in different regions and time periods [9,10]. Rotavirus vaccines are effective in preventing rotavirus infections. In Jining, there are two types of rotavirus vaccines in use: the pentavalent rotavirus vaccine (RotaTeq) (G1, G2, G3, G4, P[8]) produced by Merck, Rahway, NJ, USA, which has been used since 2018 [11,12], and the live attenuated rotavirus vaccine (LLR strain, Rotavin-M1) (G10P[15]) produced by the Lanzhou Institute of Biological Products, Lanzhou China, which has been used since 2001 [13,14]. Although rotavirus vaccines are not included in the routine immunization program in Jining, and parents need to pay for the vaccination themselves, there is a relatively high willingness among parents to vaccinate their children, with approximately 30% of infants and young children receiving the rotavirus vaccine.

NV causes approximately 680 million cases of diarrhea and leads to around 200,000 deaths annually [15]. Based on the VP1 and RdRp gene sequences of NV, it is classified into G and P genotypes. Among them, GI and GII are the primary genotypes infecting humans [16,17,18].

Global risks of viral gastroenteritis infection and outbreak persist. In China, incidents of viral gastroenteritis remain at a significant level. The characteristics of viral gastroenteritis vary across regions and time periods. Jining City, with a population of approximately 8.3 million, holds a unique position at the confluence of the Yellow River and the Grand Canal, boasting the largest freshwater lake in northern China, Weishan Lake. In this study, specimens collected from January 2021 to December 2022 in Jining City were tested and genotyped for RV-A, NV GI, and NV GII. The aim is to explore the epidemiological and genotypic features of RV and NV during this period, providing a basis for formulating prevention and control policies.

## 2. Materials and Methods

### 2.1. Sample Collection

From January 2021 to December 2022, 3–6 outpatient medical records of diarrhea cases were collected from each of the ten counties in Jining City, China, on a monthly basis. These cases met the criteria of having diarrhea (defined as three or more bowel movements per day with a change in stool consistency, fecal leukocyte count < 15, and no observed red blood cells) or exhibited symptoms of diarrhea less than three times per day, but with altered stool consistency and vomiting as a primary symptom. Fecal samples, weighing over 5 g, were then sent to the Microbiology Laboratory of Jining City Center for Disease Control and Prevention for testing. The samples were stored in a −80 °C freezer, and nucleic acid testing was completed within 30 days.

### 2.2. Nucleic Acid Testing

For each sample, 0.1 g of fecal material was mixed with 0.9 mL of PBS. This mixture underwent three rounds of vigorous shaking, followed by centrifugation at 8000× *g* for 5 min. Subsequently, 200 μL of the resulting supernatant was collected. Nucleic acid extraction was carried out using the GeneRotex 96 automatic nucleic acid extractor (GeneRotex, Xi’an Tianlong Technology Co., Ltd., Xi’an, China) in combination with the viral nucleic acid extraction reagent (T138) from the same company. Real-Time Quantitative Fluorescent Reverse Transcriptase-PCR testing for RV-A, NV GI, and NV GII was performed using the triple nucleic acid detection kit (Catalog No. A2593) provided by Beijing Zhuocheng Hui Sheng Biotechnology Co., Ltd., Beijing, China. The Real-Time Quantitative Fluorescent Reverse Transcriptase-PCR reaction conditions were as follows: 50 °C for 10 min, 95 °C for 30 s, followed by 45 cycles of 95 °C for 5 s and 60 °C for 30 s. The Real-Time Quantitative Fluorescent Reverse Transcriptase-PCR (RT-qPCR) kit from Beijing Zhuocheng Hui Sheng Biotechnology Co., Ltd. (Beijing, China) is used not only for detecting rotavirus and norovirus but also includes human ribonuclease P (RNase P), a negative control, and a positive control for quality control of nucleic acid detection. The detection results for rotavirus and norovirus are considered valid if the positive control and RNase P tests are positive, and the negative control test is negative. Additionally, the kit demonstrates a coefficient of variation of less than 5% for precision reference samples and shows no cross-reactivity with other pathogens.

### 2.3. Gene Sequencing

For RV-A-positive samples, nucleic acids were separately amplified in the VP4 region and VP7 region. The amplification primers were synthesized by Sangon Biotech (Shanghai, China) Co., Ltd. The amplification primers were as follows: VP4F: 5′-TATGCTCCAGTNAATTGG-3′; VP4R: 5′-ATTGCATTTCTTTCCATAATG-3′; VP7F: 5′-ATGTATGGTATTGAATATACCAC-3′; and VP7R: 5′-AACTTGCCACCATTTTTTCC-3′. These primers amplified nucleotides 1–887 of VP7 [19] and 133–796 of VP4 [20]. Reverse transcription and PCR amplification were performed using the HiScript One Step RT-PCR Kit (P6111) produced by Vazyme Biotech Co., Ltd., Nanjing, China. The reaction conditions were as follows: 50 °C for 30 min; 94 °C for 2 min; 94 °C for 30 s, 42 °C for 30 s, and 72 °C for 1 min, for a total of 40 cycles; and finally, 72 °C for 7 min. The amplification products were sent to Sangon Biotech (Shanghai, China) Co., Ltd. for nucleotide sequencing.

For NV-GII-positive samples, nucleic acids were amplified in the VP1 region and RdRp region. The amplification primers were synthesized by Sangon Biotech (Shanghai, China) Co., Ltd. The amplification primers were Mon431: 5′-TGGACIAGRGGICCYAAYCA-3′ and G2SKR: 5′-CCRCCNGCATRHCCRTTRTACAT-3′, amplifying nucleotides 1249–1530 of RdRp and 1–305 of VP1 [21]. Reverse transcription and PCR amplification were conducted using the HiScript One Step RT-PCR Kit (P6111) produced by Vazyme Biotech Co., Ltd., Nanjing. The RT-PCR reaction conditions were as follows: 50 °C for 30 min; 94 °C for 2 min; 94 °C for 45 s, 50 °C for 45 s, and 68 °C for 1 min, for a total of 40 cycles; and finally, 68 °C for 10 min. The amplification products were sent to Sangon Biotech (Shanghai, China) Co., Ltd. for nucleotide sequencing. Sanger sequencing was performed using the 3730XL sequencer manufactured by ABI and provided by Sangon Biotech (Shanghai, China) Co., Ltd.

### 2.4. Genotype and Phylogenetic Analysis

RV-A-positive samples underwent genotype analysis using the rotavirus A Genotype Determination software (https://www.viprbrc.org/brc/rvaGenotyper.spg?method=ShowCleanInputPage&decorator=reo) (accessed on 11 April 2023). NV-GII-positive samples were subjected to genotype analysis using the norovirus Typing Tool Version 2.0 software (https://www.rivm.nl/mpf/typingtool/norovirus/) (accessed on 11 April 2023). Reference sequences for RV-A genotypes G1P[8], G2P[4], G8P[8], G9P[8], and NV GII genotypes GII.3[P12], GII.4[P16], GII.4[P31] were downloaded from the NCBI database. After sequence alignment using MEGA 7.0.14 software [22], the neighbor-joining method was employed to construct phylogenetic trees for RV-A VP4 and VP7 genes as well as NV GII VP1 and RdRp genes. The construction of the phylogenetic tree utilized nucleotides 188–796 of the RV-A VP4 gene, nucleotides 51–875 of the VP7 gene, nucleotides 1–241 of the NV GII VP1 gene, and nucleotides 1336–1530 of the RdRp gene.

### 2.5. Statistical Analysis

Statistical analysis was conducted using SPSS 24.0 software. Inter-group comparisons were performed using the χ^2^ test, with *p* < 0.05 indicating statistically significant differences.

## 3. Results

### 3.1. Pathogen Detection

**Figure 1:**
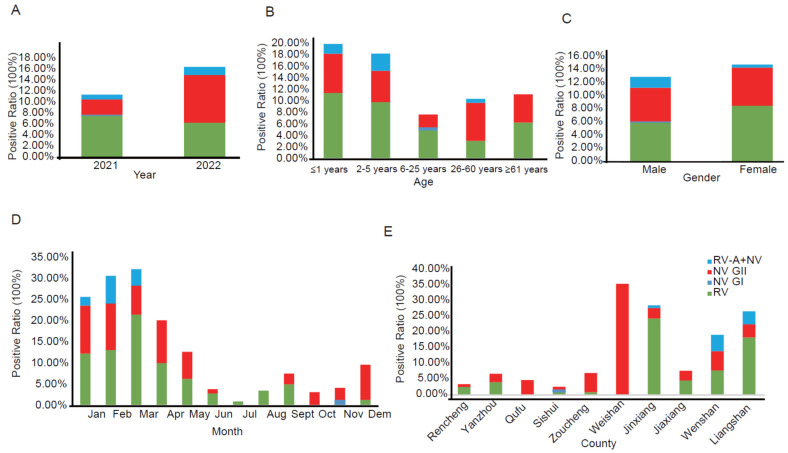
The epidemiological characteristics of norovirus and rotavirus. (**A**) Positive rates of norovirus and rotavirus in 2021 and 2022. (**B**) Infection-positive rates of norovirus and rotavirus in different age groups. (**C**) Infection-positive rates of norovirus and rotavirus in different genders. (**D**) Infection-positive rates of norovirus and rotavirus in different months. (**E**) Infection-positive rates of norovirus and rotavirus in different counties. “■” RV-A, “■” NVGI, “■” NVGII, “■” RV-A + NV GII.

RV-A and NV were identified in both 2021 and 2022. In 2021, the overall positivity ratio for RV-A and NV was 11.42% (66/578), whereas in 2022, the ratio increased to 16.46% (78/474). The detection ratio for RV-A (excluding co-infection of RV-A and NV GII, subsequent mentions of RV-A positivity will also exclude co-infection of RV-A and NV GII) was 7.03% (74/1052) and for NV GII (excluding co-infection of RV-A and NV GII, subsequent mentions of NV GII positivity will also exclude co-infection of RV-A and NV GII) was 5.42% (57/1052). These ratios were higher in both 2021 and 2022 compared to NV GI, which had a detection ratio of 0.10% (1/1052). Additionally, 1.14% (12/1052) of the samples showed the presence of RV-A together with NV GII. The difference in RV-A detection ratios between 2021 (7.61%, 44/578) and 2022 (6.33%, 30/474) was not statistically significant (χ^2^ = 0.66, *p* = 0.42). In 2022, the NV GII detection rate (8.65%, 41/474) was higher than in 2021 (2.77%, 16/578) (χ^2^ = 17.58, *p* < 0.001) (Table 1, Figure 1A).

The detection ratios of RV-A varied across different age groups. Among the ≤1 years and 2–5 years age groups, the detection ratios for RV-A were relatively higher at 11.44% (27/236) and 9.85% (20/203), respectively. In contrast, the 6–25 years and 26–60 years age groups showed lower detection ratios for RV-A, at 4.95% (9/182) and 3.12% (9/288), respectively (χ^2^ = 17.54, *p* < 0.001). NV GII had higher detection ratios in both the ≤1 years and 26–60 years age groups compared to the 6–25 years age group (χ^2^ = 4.74, *p* = 0.03, χ^2^ = 4.64, *p* = 0.03). Co-infections of RV-A and NV GII had higher detection ratios in the ≤1 years and 2–5 years age groups, at 1.69% (4/236) and 2.96% (6/203), respectively, while they were not detected in the 6–25 years or ≥61 years age groups (χ^2^ = 10.83, *p* = 0.03) (Table 1, Figure 1B).

There were no statistically significant differences in the detection ratios of RV-A (χ^2^ = 2.56, *p* = 0.11) and NV GII (χ^2^ = 0.24, *p* = 0.62) between males and females. Similarly, there was no statistically significant difference in the detection ratios of RV-A and NV GII co-infections between males and females (χ^2^ = 3.13, *p* = 0.07) (See Table 1, Figure 1C).

In different months, the detection ratios of RV-A were higher in January to April, with ratios of 12.37% (12/97), 13.19% (12/91), 21.57% (22/102), and 10.10% (10/99), respectively, while in June–July and October to December, the detection ratios were lower, at 2.94% (3/102), 1.01% (1/99), 0 (0/62), 0 (0/71), and 1.39% (1/72), respectively (χ^2^ = 67.62, *p* < 0.001). From December to May, the NV GII detection ratios were higher, at 8.33% (6/72), 11.34% (11/97), 10.99% (10/91), 6.86% (7/102), 10.10% (10/99), 6.38% (6/94), while in July to November, the NV GII detection ratios were lower, at 0.98% (1/102), 0 (0/99), 0 (0/84), 2.53% (2/79), 3.23% (2/62), 2.82% (2/71) (χ^2^ = 35.37, *p* < 0.001). RV-A and NV GII co-infections were only detected in January to March, with detection ratios of 2.06% (2/97), 6.59% (6/91), and 3.92% (4/102), while they were not detected from April to December (χ^2^ = 40.51, *p* < 0.001) (See Figure 1D).

Jinxiang County and Liangshan County exhibited higher RV-A detection ratios at 24.37% (29/119) and 18.33% (22/120), respectively. Conversely, Qufu, Weishan, Sishui, and Zoucheng displayed lower RV-A detection ratios, registering 0% (0/85), 0% (0/62), 0.86% (1/116), and 0.88% (1/114), respectively (χ^2^ = 108.46, *p* < 0.001). Weishan showed the highest NV GII detection rate at 35.48% (22/62), while Rencheng, Sishui, and Yanzhou had lower NV GII detection ratios, measuring 0.85% (1/117), 0.86% (1/116), and 2.70% (2/74), respectively (χ^2^ = 122.92, *p* < 0.001). RV-A and NV GII co-infections were only observed in Wenshan, Liangshan, and Jinxiang, with detection ratios of 5.22% (6/115), 4.17% (5/120), and 0.84% (1/119), respectively. They were not detected in Rencheng, Yanzhou, Qufu, Sishui, Zoucheng, Weishan, or Jiaxiang (χ^2^ = 34.84, *p* < 0.001) (See Figure 1E).

### 3.2. RV-A Genotype Analysis

**Figure 2:**
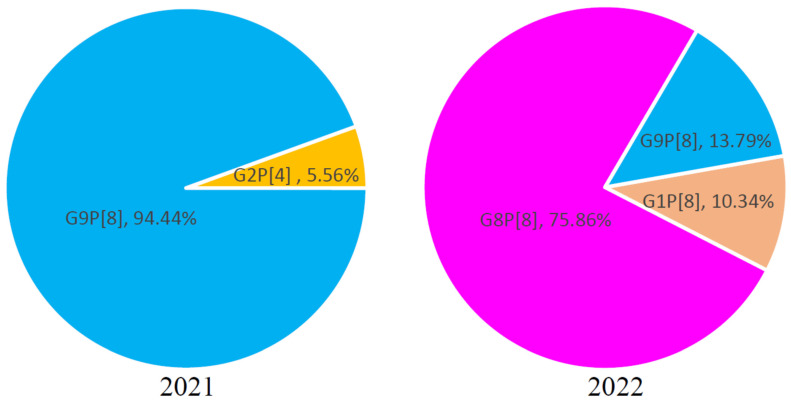
Genotype distribution of RV-A in Jining City from 2021 to 2022. Left figure: proportional distribution of different genotypes of RV-A in Jining City in 2021. Right figure: proportional distribution of different genotypes of RV-A in Jining City in 2022. “■” G9P[8], “■” G8P[8], “■” G2P[4], “■” G1P[8].

To determine the genotypes of RV-A in Jining City from 2021 to 2022, sequencing of the VP7 and VP4 genes was conducted on RV-A-positive samples, resulting in a total of 47 sequences. According to the VP7 gene typing, also known as G genotypes, in the 47 sequences collected from Jining City between 2021 and 2022, the G1 genotype accounted for 6.38% (3/47), G2 genotype accounted for 2.13% (1/47), G8 genotype accounted for 46.81% (22/47), and G9 genotype accounted for 44.68% (21/47). No G3 or G4 genotypes were detected. Based on the VP4 gene typing, also known as P genotypes, in the 47 sequences from Jining City between 2021 and 2022, the P[4] genotype accounted for 2.13% (1/47), and the P[8] genotype accounted for 97.87% (46/47). No P[6] genotypes were found (Figure 2).

In 2021, the G2P[4] genotype of RV-A in Jining City accounted for 5.56% (1/18), while the G9P[8] genotype accounted for 94.44% (17/18). This indicates that the prevalent RV-A genotype in Jining City in 2021 was primarily G9P[8], co-circulating with the G2P[4] genotype. In 2022, the G1P[8] genotype of RV-A in Jining City accounted for 10.35% (3/29), the G8P[8] genotype accounted for 75.86% (22/29), and the G9P[8] genotype accounted for 13.79% (4/29) (Figure 2). This suggests that the prevalent RV-A genotypes in Jining City shifted to predominantly G8P[8] in 2022, with concurrent circulation of G1P[8] and G9P[8] genotypes. This study indicates that there may have been a change in prevalent RV-A genotypes between 2021 and 2022 in Jining City.

### 3.3. RV-A Genomic Tree Analysis

The main genotypes of RV-A in Jining City from 2021 to 2022 were G1P[8], G2P[4], G8P[8], and G9P[8]. Reference sequences of G1P[8], G2P[4], G8P[8], and G9P[8] genotypes from 2016 to 2022 were obtained from the GenBank database to construct the evolutionary tree.

**Figure 3:**
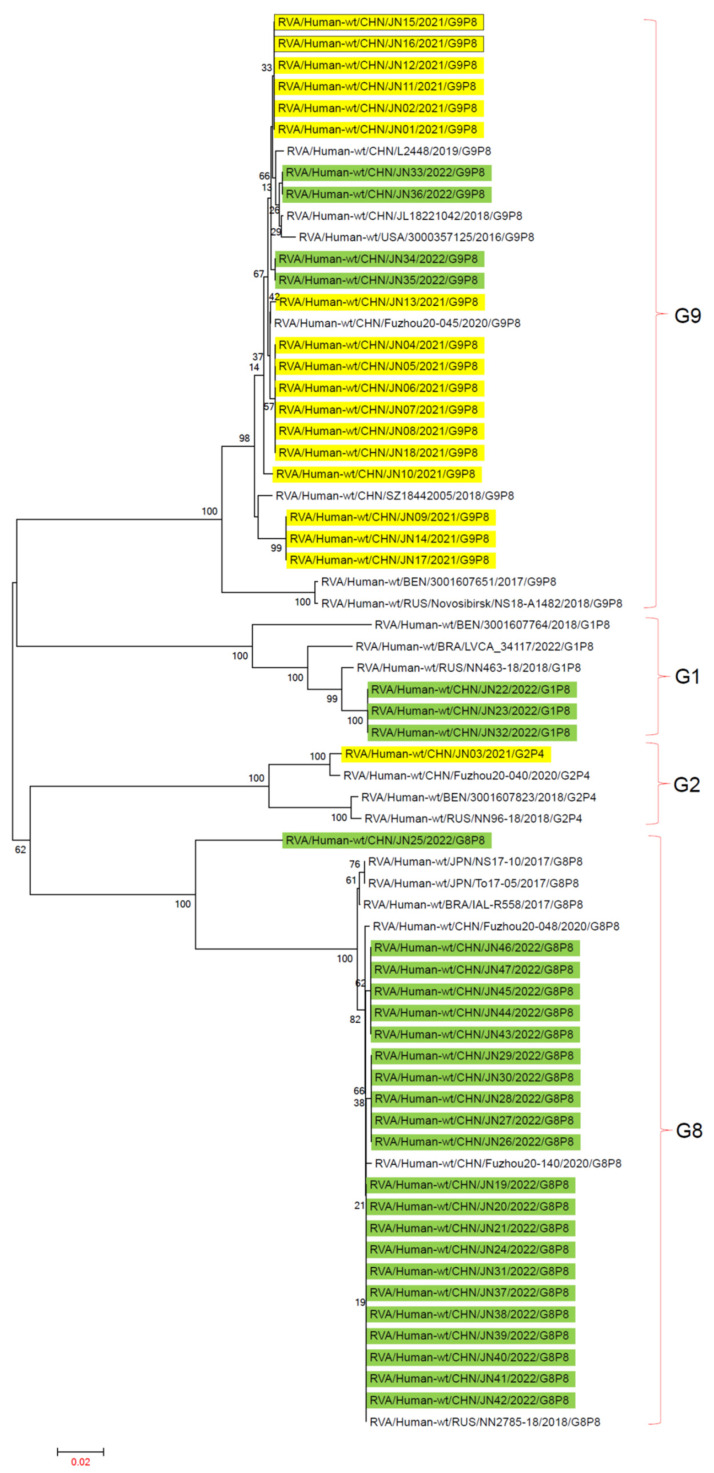
Phylogenetic trees based on VP7 nucleotide sequences of RV-A. An evolutionary tree was constructed using 47 rotavirus VP7 gene sequences from Jining City and 19 rotavirus VP7 gene sequences downloaded from NCBI from recent years, using the maximum-likelihood method with 1000 bootstrap replicates in the MEGA 7.0.14 software. The RV-A G genotype analysis was conducted using the rotavirus A Genotype Determination software, with reference sequences sourced from NCBI. “■” represents RV-A in 2021, “■” represents RV-A in 2022.

In the VP7 gene evolutionary tree, G1, G2, G8, and G9 genotypes were located on four different evolutionary branches. In the G1 evolutionary branch, the three G1P[8] genotypes from Jining City in 2021–2022 independently formed one evolutionary cluster. One G2P[4] genotype from Jining City clustered with China’s Fuzhou20-40. In the G8 evolutionary branch, the G8P[8] genotypes from Jining City were distributed in two evolutionary clusters. Among them, JN25 formed an independent evolutionary cluster, while the other 21 G8P[8] genotypes were in the same cluster as China’s Fuzhou20-140, NN2785-18, and others. In the G9 evolutionary branch, JN09, JN14, and JN17 were in separate evolutionary clusters, while the other 18 G9 genotypes from Jining City were in another cluster (Figure 3).

**Figure 4:**
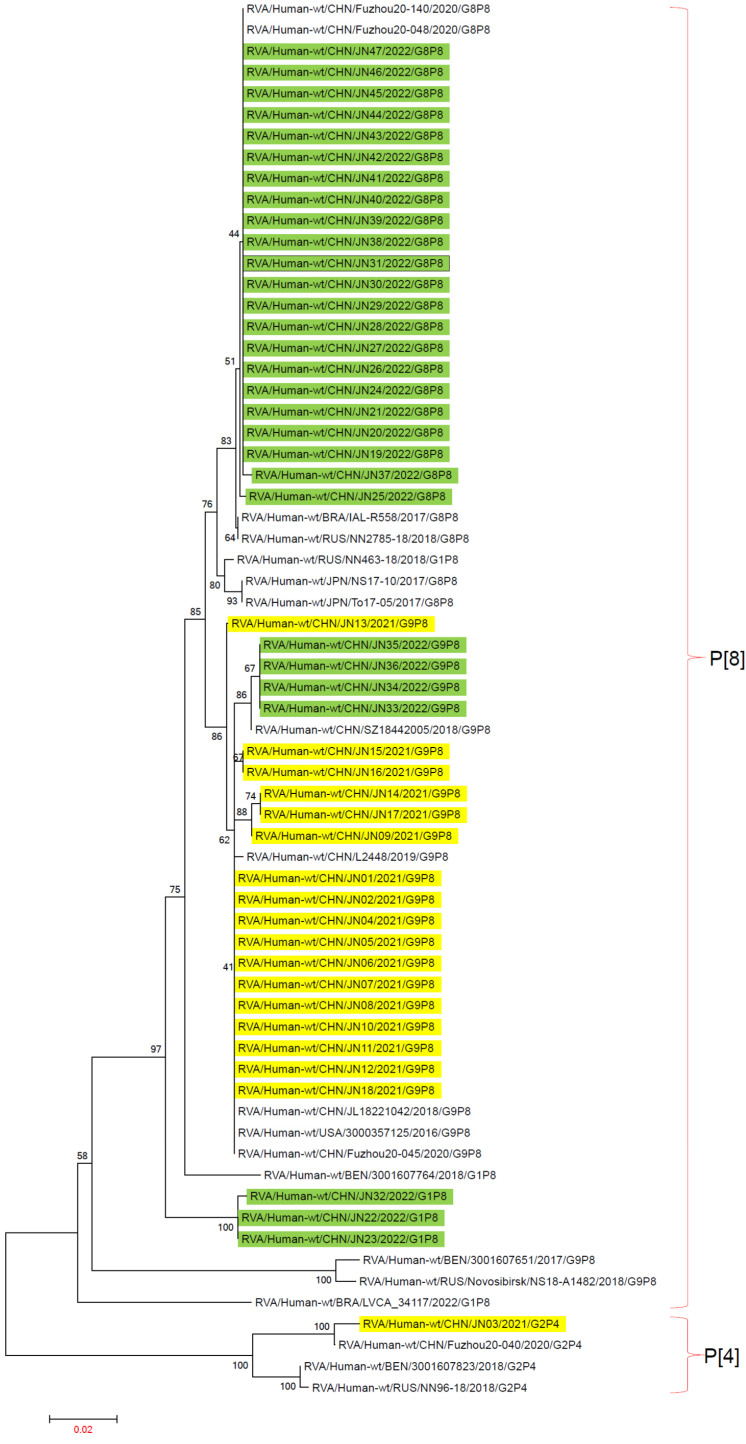
Phylogenetic trees based on VP4 nucleotide sequences of RV-A. An evolutionary tree was constructed using 47 rotavirus VP4 gene sequences from Jining City and 19 rotavirus VP4 gene sequences downloaded from NCBI from recent years using the maximum-likelihood method with 1000 bootstrap replicates in MEGA 7.0.14 software. “■” represents RV-A in 2021, and “■” represents RV-A in 2022. RV-A P genotype analysis was performed using the rotavirus A Genotype Determination software, with reference sequences obtained from NCBI.

In the VP4 gene evolutionary tree, P[4] and P[8] genotypes were located on two different evolutionary branches. In the P[4] evolutionary branch, the G2P[4] genotype JN03 from Jining City clustered with China’s Fuzhou20-040. In the P[8] evolutionary branch, the three G1P[8] genotypes from Jining City formed separate clusters, showing distant genetic affinity to the reference G1P[8] genotype sequences. The G8P[8] genotype sequences from Jining City all clustered together and were closely related to G8P[8] genotypes from China’s Fuzhou20-048, Fuzhou20-140, Brazil’s IAL-R558, and Russia’s NN2785-18. The G9P[8] genotype sequences from Jining City also clustered together, showing genetic affinity to G9P[8] genotypes from China’s Fuzhou20-045, USA’s 3000357125, and others, but were distantly related to the G9P[8] genotypes from Russia’s Novosibirsk NS18-A1482, the Republic of Benin’s 3001607651, and others, which formed a separate evolutionary cluster (Figure 4).

### 3.4. Genotype Analysis of NV GII

To determine the genotypes of NV GII in Jining City from 2021 to 2022, we sequenced the VP1 and RdRp regions of NV-GII-positive samples, successfully obtaining 27 viral sequences.

**Figure 5:**
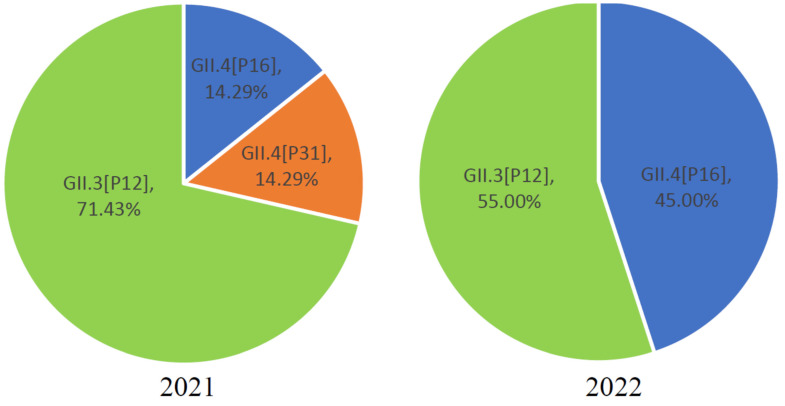
The genotype distribution of NV GII in Jining City from 2021 to 2022. Left figure: proportional distribution of different genotypes of NV GII in Jining City in 2021. Right figure: proportional distribution of different genotypes of NV GII in Jining City in 2022. “■” represents GII.3[P12], “■” represents GII.4[P16], and “■” represents GII.4[P31].

In the sequences obtained from Jining City in 2021–2022, GII.3 genotypes accounted for 59.26% (16/27), while GII.4 genotypes accounted for 40.74% (11/27). Other genotypes, such as GII.1, GII.2, GII.5-GII.10, and GII.20-GII.27, were not detected. This indicates that the predominant genotypes of NV GII in Jining City from 2021 to 2022 were GII.3 and GII.4. Among the sequences obtained from Jining City in 2021–2022, GII.P12 accounted for 59.26% (16/27), GII.P16 accounted for 37.04% (10/27), and GII.P31 accounted for 3.70% (1/27). Other genotypes like GII.P1-GII.8 and GII.P20-GII.P41 were not detected. This indicates that the prevalent genotypes of NV GII in Jining City from 2021 to 2022 were GII.P12, GII.P16, and GII.P31 (Figure 5).

In 2021, among the NV GII in Jining City, GII.3[P12] accounted for 71.42% (5/7), GII.4[P16] accounted for 14.29% (1/7), and GII.4[P31] accounted for 14.29% (1/7). This suggests that in 2021, the predominant NV GII genotype in Jining City was GII.3[P12], with co-circulation of GII.4[P16] and GII.4[P31]. In 2022, among the NV GII in Jining City, GII.3[P12] accounted for 55.00% (11/20), while GII.4[P16] accounted for 45.00% (9/20) (Figure 5). This indicates that in 2022, the GII.3[P12] and GII.4[P16] genotypes of NV GII co-circulated in Jining City.

### 3.5. Evolutionary Tree Analysis of NV GII Genotypes

In Jining City, the main genotypes of NV GII in 2021–2022 were GII.3[P12], GII.4[P16], and GII.4[P31]. Genotype sequences from 2016 to 2022, including GII.3[P12], GII.4[P16], and GII.4[P31], were obtained from the GenBank database to construct a reference tree.

**Figure 6:**
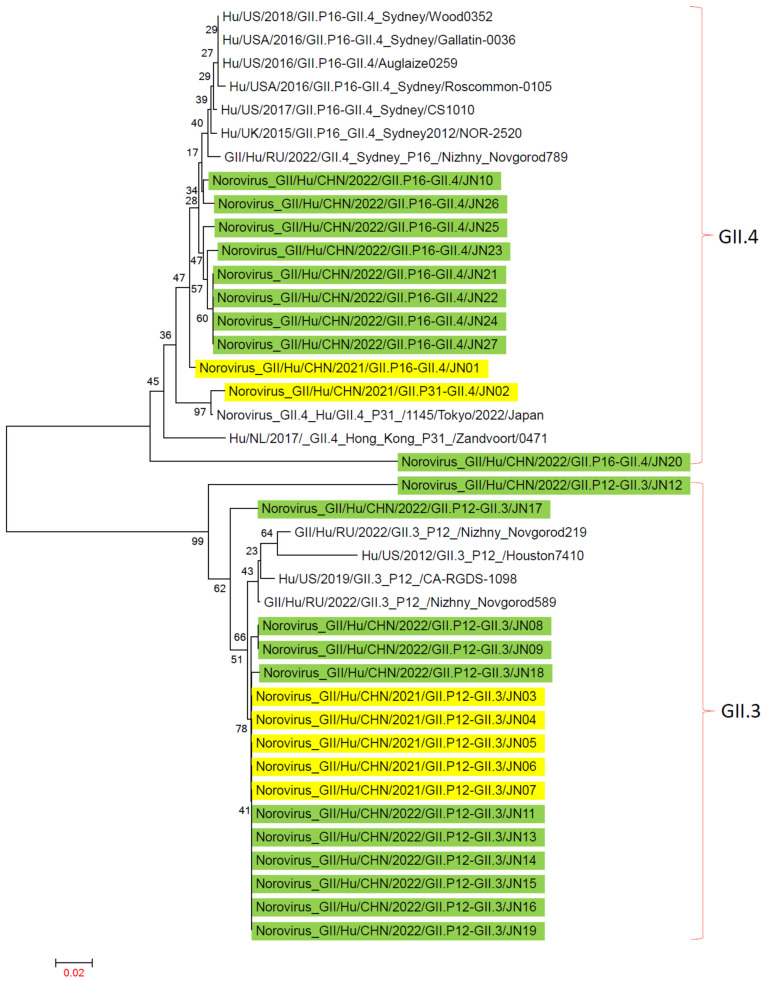
Phylogenetic trees based on VP1 nucleotide sequences of NV GII. An evolutionary tree was constructed using 27 NV GII VP1 gene sequences from Jining City and 13 NV GII VP1 sequences downloaded from NCBI from recent years using the maximum-likelihood method with 1000 bootstrap replicates in MEGA 7.0.14 software. NV GII G genotype analysis was conducted using the norovirus Typing Tool Version 2.0 software, with reference sequences sourced from NCBI. “■” represents NV GII in 2021, and “■” represents NV GII in 2022.

In the evolutionary tree based on the VP1 gene, the GII.3 and GII.4 genotypes were located on two different evolutionary branches. On the GII.3 branch, JN12 and JN17 of the GII.3[P12] genotype from Jining City formed one evolutionary cluster, while the other 14 strains of GII.3[P12] from Jining City clustered separately. On the GII.4 branch, strains from Jining City were distributed among three different evolutionary clusters. Strain JN02 of the GII.4[P31] genotype from Jining City clustered with similar strains from Tokyo, Japan. Among the 10 strains of the GII.4[P16] genotype from Jining City, JN20 was grouped in one cluster, while the other nine strains formed a separate cluster (Figure 6).

**Figure 7:**
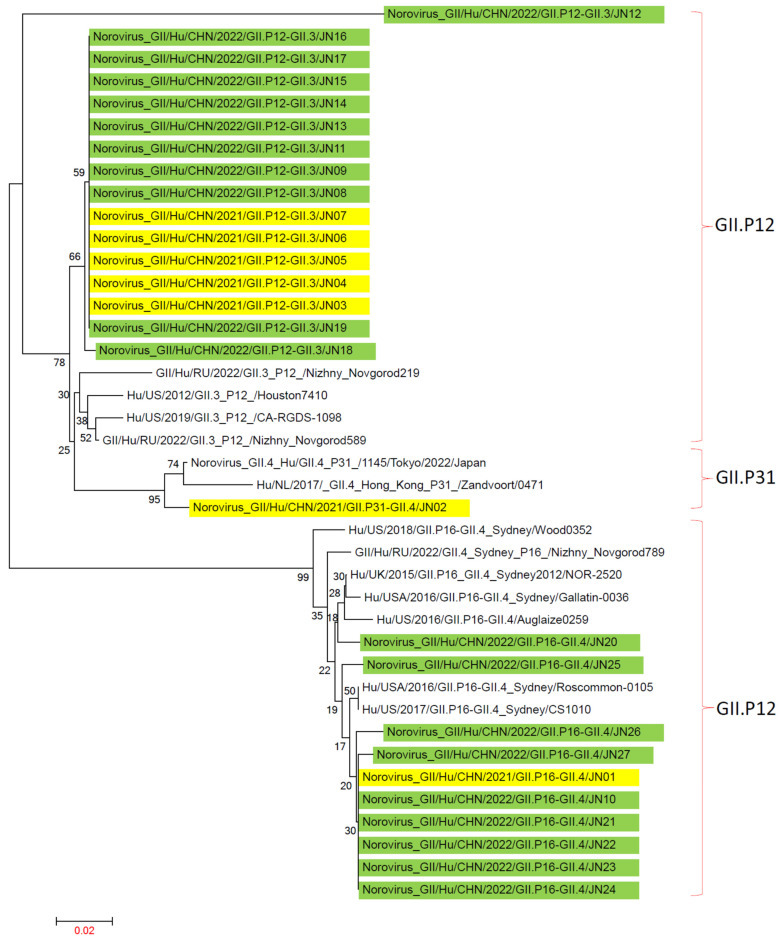
Phylogenetic trees based on RdRp nucleotide sequences of NV GII. An evolutionary tree was constructed using 27 NV GII RdRp gene sequences from Jining City and 13 NV GII RdRp sequences downloaded from NCBI from recent years using the maximum-likelihood method with 1000 bootstrap replicates in the MEGA 7.0.14 software. NV GII P genotype analysis was performed using the norovirus Typing Tool Version 2.0 software, with reference sequences obtained from NCBI. “■” represents NV GII in 2021, and “■” represents NV GII in 2022.

In the RdRp-based evolutionary tree, GII.P12, GII.P16, and GII.P31 genotypes were located on distinct evolutionary branches. On the GII.P12 branch, strain JN12 of the GII.3[P12] genotype from Jining City formed an independent evolutionary cluster, while the other 15 strains of GII.3[P12] from Jining City clustered separately. On the GII.P16 branch, JN20 was grouped in one cluster, while the other nine strains of the GII.4[P16] genotype from Jining City formed a separate cluster. On the GII.P31 branch, strain JN02 from Jining City and similar strains from Tokyo, Japan, clustered together (Figure 7). This study suggests that NV GII of the same genotype may have different origins and evolutionary pathways.

## 4. Discussion

The harm caused by viral infection to the human body is significant [23]. This study presents a detailed two-year systematic investigation of the infection ratios and genotypic characteristics of RV-A and NV in diarrheal cases in Jining City, Northern China. The findings reveal that RV-A and NV collectively accounted for 13.69% of diarrheal cases, which is lower than the positivity ratios reported in the Southwest region of China from 2018 to 2020 [24]. Specifically, the RV-A positivity rate in Jining City was 7.03%, a figure similar to the 7.22% reported in Beijing, Northern China, from 2018 to 2022 [25]. This rate is, however, lower than the 15.63% positivity rate observed in Wuhan, Central China, from 2019 to 2022 [26]. This contrasts with broader studies which indicate more consistent detection ratios across different age groups, illustrating the variability in RV-A epidemiology across different settings. However, it should be noted that the RV-A detection kit used in this study cannot distinguish between vaccine strains and wild strains, which may affect the detection results. In Jining City, the positivity rate for NV is 5.51%, predominantly attributed to NV GII, while the positivity rate for NV GI is only 0.10%. This distribution aligns with the prevalent results of NV genotypes in China. The positivity rate for NV GII in Jining City is lower compared to the Southwest region of China from 2018 to 2020 but is in line with findings from Wuhan, China [24,25,27].

In China, RV-A and NV show distinct seasonal patterns, with RV-A more prevalent from December to May, and NV exhibiting a higher incidence from October to May [24]. This study in Jining City found that RV-A had the highest detection ratios from January to April, while NV peaked from December to May.

These trends were consistent with established patterns in Northern China. There were significant differences in detection ratios among counties in Jining City. For example, Jinxiang County had a high RV-A detection rate of 24.37%, whereas Weishan and Qufu counties showed no RV-A detection. Weishan County stood out with a remarkably high NV GII detection rate of 35.48%, while other counties had ratios below 6.14%. This emphasizes how distinct county environments impact RV-A and NV GII prevalence. Notably, Weishan County hosts one of Northern China’s largest freshwater lakes, Weishan Lake, which is a major source of freshwater products in China. It produces over ten types of shellfish and various freshwater fish species. There may be norovirus contamination in these freshwater products, and improper consumption of these products is one of the significant causes of norovirus infections, likely influencing the distribution of RV-A and NV [28,29]. Additionally, a significant number of diarrheal cases involved mixed RV-A and NV GII infections [24,30]. This study identified a substantial number of such cases, and their distribution mirrors patterns observed for norovirus and rotavirus in terms of age, month, and county.

RV-A can be classified into different G and P genotypes based on its VP7 and VP4 gene sequences. In Asia, the predominant genotypes of RV-A have shown variation over the years [10,25]. From 2009 to 2013, G1P[8] and G2P[4] were prevalent. This shifted to G8P[8] in 2014–2015 [10]. Between 2016 and 2020, the main genotypes became G9P[8] and G3P[8], and post-2021, they further transitioned to G9P[8] and G8P[8] [10,25,31,32,33]. This study successfully identified the G and P genotypes of 47 RV-A viruses, and the results were in agreement with the phylogenetic tree analysis. In 2021, G9P[8] was predominant, co-circulating with G2P[4]. In 2022, G8P[8] emerged as the predominant genotype, alongside G9P[8] and G1P[8]. This indicates that although G9P[8] and G8P[8] have been predominant in Asia in recent years, other genotypes still coexist, in line with findings in Beijing and Wuhan, China [25,26]. The G8P[8] genotype of RV-A began to emerge in China in 2020–2021, and by 2022, it became the prevailing genotype, accounting for a high proportion of 75.86%. This mirrors results in Beijing and Wuhan, suggesting that G8P[8] is currently the dominant genotype in China. In the phylogenetic trees based on VP7 and VP4 genes, the G8P[8] genotype RV-A in Jining City is closely related to strains from Fuzhou, a city in southern China, in 2020. This suggests that the circulating G8P[8] genotype RV-A in China may have a common or closely related origin. Moreover, RV-A vaccines have proven effective in preventing RV-A infections [34,35]. In Jining, China, two types of rotavirus vaccines are used: the RotaTeq rotavirus vaccine, which contains the G1, G2, and P[8] genotypes, and the LLR strain vaccine [11,12]. The use of rotavirus vaccines in infants and young children in Jining may provide immunological protection and reduce infections, particularly from genotypes G1, G2, G3, G4, and P[8]. Additionally, considering the complex diversity of rotavirus genotypes, both domestic and imported rotavirus vaccines should consider multivalent vaccines and include genotypes G8, G9, and P[8] to promote better protection against G8P[8] and G9P[8] genotype rotaviruses.

Based on the VP1 and RdRp gene sequences of NV, it can be classified into different G and P genotypes. Since 2015, the predominant genotypes in China have been GII.2[P16], GII.3[P12], GII.4[P16], and GII.4[P31] [27,36,37,38,39]. In 2021 and 2022, the predominant genotypes in Jining City were GII.3[P12], co-circulating with GII.4[P16] and GII.4[P31]. GII.2[P16] and other genotypes were not detected in Jining City, which may be attributed to the limited sample size and the concentration of NV cases in Weishan County in this study. Additionally, in the phylogenetic trees based on VP1 and RdRp genes, both GII.3[P12] and GII.4[P16] genotypes in Jining City exhibited two distinct evolutionary clusters. This suggests that the VP1 gene of the GII.3[P12] and GII.4[P16] genotypes in Jining City may have at least two different evolutionary sources.

It should be noted that in September 2022, Jining City experienced an outbreak of the novel coronavirus. Due to strict local COVID-19 control measures, the collection of diarrheal specimens in some counties was affected, leading to a lower sample volume during this period. As a result, the final analysis results may have potential biases.

In summary, RV-A and NV GII are the primary pathogens causing diarrheal cases in Jining City, with a higher incidence in the winter and spring seasons. The prevalence of RV-A and NV GII varies among different counties. From 2021 to 2022, the predominant genotype of RV-A shifted from G9P[8] to G8P[8], while the dominant genotypes of NV GII remained unchanged over these two years. It is crucial to strengthen long-term monitoring and genotype analysis of RV-A and NV in various regions of China, providing valuable data for the development of new vaccine strains. Additionally, tailored vaccination strategies should be devised for different counties and age groups to more effectively mitigate the risk of RV-A and NV infections.

## Figures and Tables

**Table 1:** Distribution characteristics of rotavirus and norovirus in outpatient diarrhea-positive cases in Jining City, 2021–2022.

	Sample Size	Positive Cases (Positivity Ratios %)				
		RV-A	NV GI	NV GII	RV-A + NV GII	Total
Year						
2021	578	44 (7.61)	1 (0.17)	16 (2.77)	5 (0.87)	66 (11.42)
2022	474	30 (6.33)	0 (0)	41 (8.65)	7 (1.48)	78 (16.46)
Age						
≤1	236	27 (11.44)	0 (0)	16 (6.78)	4 (1.69)	47 (19.92)
2–5	203	20 (9.85)	0 (0)	11 (5.42)	6 (2.96)	37 (18.23)
6–25	182	9 (4.95)	1 (0.55)	4 (2.20)	0 (0)	14 (7.69)
26–60	288	9 (3.12)	0 (0)	19 (6.60)	2 (0.69)	30 (10.42)
≥61	143	9 (6.29)	0 (0)	7 (4.90)	0 (0)	16 (11.19)
Gender						
Male	605	36 (5.95)	1 (0.17)	31 (5.12)	10 (1.65)	78 (12.89)
Female	447	38 (8.50)	0 (0)	26 (5.82)	2 (0.45)	66 (14.77)
Total	1052	74 (7.03)	1 (0.10)	57 (5.42)	12 (1.14)	144 (13.69)

## Data Availability

The datasets have been submitted to GenBank. The accession numbers are PRJNA1114740 and PRJNA1114741. The data that support the findings of this study are available from the corresponding author upon reasonable request.

## Abbreviations

RT-PCR	Reverse Transcription—Polymerase Chain Reaction
RV	rotavirus
NV	norovirus
